# Validation of the Social Networking Activity Intensity Scale among Junior Middle School Students in China

**DOI:** 10.1371/journal.pone.0165695

**Published:** 2016-10-31

**Authors:** Jibin Li, Joseph T. F. Lau, Phoenix K. H. Mo, Xuefen Su, Anise M. S. Wu, Jie Tang, Zuguo Qin

**Affiliations:** 1 Division of Behavioral Health and Health Promotion, The Jockey Club School of Public Health and Primary Care, Faculty of Medicine, The Chinese University of Hong Kong, Hong Kong, China; 2 Department of Clinical Research, Sun Yat-sen University Cancer Center, State Key Laboratory of Oncology in South China, Collaborative Innovation Center for Cancer Medicine, Guangzhou, China; 3 The Chinese University of Hong Kong Shenzhen Research Institute (SZRI), Shenzhen, China; 4 Department of Psychology, Faculty of Social Sciences, University of Macau, Macao, China; 5 Center for Health Education in Guangdong Province, Guangdong, China; University of the Basque Country, SPAIN

## Abstract

**Background:**

Online social networking use has been integrated into adolescents’ daily life and the intensity of online social networking use may have important consequences on adolescents’ well-being. However, there are few validated instruments to measure social networking use intensity. The present study aims to develop the Social Networking Activity Intensity Scale (SNAIS) and validate it among junior middle school students in China.

**Methods:**

A total of 910 students who were social networking users were recruited from two junior middle schools in Guangzhou, and 114 students were retested after two weeks to examine the test-retest reliability. The psychometrics of the SNAIS were estimated using appropriate statistical methods.

**Results:**

Two factors, Social Function Use Intensity (SFUI) and Entertainment Function Use Intensity (EFUI), were clearly identified by both exploratory and confirmatory factor analyses. No ceiling or floor effects were observed for the SNAIS and its two subscales. The SNAIS and its two subscales exhibited acceptable reliability (Cronbach’s alpha = 0.89, 0.90 and 0.60, and test-retest Intra-class Correlation Coefficient = 0.85, 0.87 and 0.67 for Overall scale, SFUI and EFUI subscale, respectively, *p*<0.001). As expected, the SNAIS and its subscale scores were correlated significantly with emotional connection to social networking, social networking addiction, Internet addiction, and characteristics related to social networking use.

**Conclusions:**

The SNAIS is an easily self-administered scale with good psychometric properties. It would facilitate more research in this field worldwide and specifically in the Chinese population.

## Introduction

Globally, Internet-based technology has increased dramatically during the past decade. Online social networking, as a new phenomenon, allows users to create individual public profiles and share information about himself/herself with a large number of people.[1] Over 70% of the teenagers in the U.S. are users of social media.[2] The annual report of the China Internet Network Information Center estimated that there were 618 million Internet users, or “netizens” (45.8% of the total population) in China at the end of 2013, and a quarter (24.1%) of them were between 10–19 years old.[3] Of these “netizens”, 48.8% were social media users, while 28.8% of the social media users were between 10–19 years old (2012).[3,4] Furthermore, previous studies conducted in China have reported prevalence of Internet addiction ranging from 2.4% to 22.9% among adolescents.[5]

With the high penetration rate, it is important to examine reasons and health outcomes of online social networking among adolescents. The literature has suggested that many adolescents use online social networking mainly for interactions with their “offline friends”. [6] However, the directions of the associations between intensity of social networking use and health outcomes are inconsistent. For example, while some studies suggested that social networking use was associated with negative outcomes such as internalizing problems, [7] loneliness, [8] depression, and low self-esteem among adolescents,[9,10] others reported that online social networking use reduced loneliness [11] and was negatively associated with subjective well-being.[12] Furthermore, studies conducted among college students found no significant association between amount of time spent on social networking and depression.[13,14]

To gauge the impact of online social networking use among adolescents, it is warranted to develop validated instruments to assess online social networking use intensity (SNUI). Several such scales exist but they have some serious limitations. First, the majority of them assessed online social networking use by simple or single-item measures (e.g. number of Facebook friends [15], average daily time spent on social networking [10], and frequency of use [16]), which fails to assess complex constructs of online social networking use. Second, although some specific scales assessed intensity of online social networking use, [17–19] they tended to focus only on a single online social networking platform (e.g. Facebook). For instance, the Facebook Intensity Scale, [17] a commonly used instrument, [8,9,20] assesses the extent of one’s emotional connection to Facebook and integration between its use and one’s daily life. It only uses one item to assess intensity of social networking use. Furthermore, it only assesses attitudes towards Facebook but no other social networking platforms, while the majority of adolescents use multiple types of social media. [21] Furthermore, the study provided information on internal consistency without mentioning other psychometric properties. [17] Third, those measures failed to capture the intensity of the full range of online activities. For instance, a study from Yang *et al* [22] only covered four specific types of Facebook activities (electronic interactions, voyeurism, self-presentation, and gaming), while intensity of using two of these four types of activities was only assessed by a single item. This is largely inadequate as adolescents usually engage in multiple types of social networking activities [6,22–24] (e.g. messaging with friends, posting comments, status updating). It is warranted to develop a more comprehensive tool to assess intensity of online social networking use, considering various types of online social networking activities, functions and platforms.

In the present study, SNUI is defined as self-reported frequency of using multiple types of online social networking activities through multiple types of platforms in the last month. We developed a new tool, the Social Networking Activity Intensity Scale (SNAIS), which can be used to assess SNUI among Chinese junior middle school students. We examined its psychometric properties including internal consistency, test-retest reliability, and construct validity. We hypothesized that the SNAIS score would be correlated with external variables such as emotional connection to social networking, social networking addiction, Internet addiction and general characteristics of social networking use (e.g. duration of social networking use).

## Methods

### Participants

The survey was conducted in October of 2013 in Guangzhou, the capital city of Guangdong province located in southern China. The population size of the city was about 12.8 million (2012). [25] Two junior middle schools, one from rural and one from urban areas, were conveniently selected to join the study, with all grade 7–9 classes of the rural school and all grade 7–8 classes of the urban school being invited to participate in the study. There were a total of 11-grade 7 classes, 11-grade 8 classes, and 5-grade 9 classes. The study hence used a convenience sampling design. All students of these selected classes were invited to participate in the study. With verbal informed consent, 1088 students participated in the study, representing a response rate of 93.3%. As a measure of quality control, 73 (6.7%) of the 1088 collected questionnaires with at least one measurement scale having >20% of its items missing were excluded from data analysis. Data obtained from 910 (89.7%) students, who were social networking users, were used for data analysis. To gauge test-retest reliability, 114 students from the school in the urban area completed the same questionnaire after two weeks.

### Procedure

An anonymous structured questionnaire was self-administered by the participants in the absence of teachers and in classroom settings, supervised by a well-trained field-worker of our research team. School consent and permission for the in-school survey was approved by school principals before the survey. We did not try to get the informed consent from parents or caregivers of the potential students considering that the survey was conducted in schools. It is thought that the school principals were responsible for students during the school time, and their informed consent would be enough on behalf of junior middle school students for the school-based study. However, the leaflets with significance and logistics of the study was sent to the school principals and potential students together before several days of conducting the survey, and the students were suggested to approach the leaflets to their parents/caregivers for the information purpose as possible. Besides, Verbal informed consent was also obtained from participants before they filled out the questionnaires by the field-workers, and it is thought that their participation or not would partially reflect themselves and their parents’ (or caregivers’) decision. Moreover, information on the study’s background and purpose, and confidentiality of the study was printed on the cover page of the questionnaire. The nature of voluntary participation was clearly mentioned in an announcement and students were informed that they had a right to terminate the study at any time. In order to maximally ensure confidentiality and protect participants from personal information disclosure, the survey applied anonymous approach, and the data analyzed anonymously. Our survey did not involve endangered or protected species, and the study did not involve sensitive content related to students.

No incentive was given to the students. The study and corresponding consent procedure was approved by the Survey and Behavioral Research Ethics Committee of the Chinese University of Hong Kong.

### Development of the social networking activity intensity scale (SNAIS)

A literature review on online activities and related measures on social networking use was conducted. [6,22,26,27] A pool of 38 items was extracted. A panel consisting of a behavioral scientist, two psychologists and an epidemiologist held several meetings and eliminated the overlapping items while combining others with similar meanings. The 14 remaining items were written as questions:” How often have you performed the following online social networking activities in the last month?” Item response scale included six categories: 0 (never), 1 (few), 2 (occasional), 3 (sometimes), 4 (often) and 5 (always). The 14-item scale was pilot tested among 77 secondary school students who were social networking users. All students understood the items well and found the questions not difficult to answer. Four additional students answered an open-ended question on whether there were any other types of online activities unmentioned. Answers obtained included “chatting with strangers”, “online shopping”, and “reading/searching materials”. These suggested activities overlapped with the current 14 items (see Table 1). In order to further refine the existing items and response scale, individual interviews were conducted among 20 students. The interviewees expressed that the 14 items captured the common functions and activities of online social networking use and were suitable to the Chinese context. Based on their comments, we removed the item response category of ‘occasional’, and thus a 5-point scale was used.

**Table 1:** Description of sample characteristics (*n* = 910).

	Total (*n* = 910)	Female (*n* = 371)	Male (n = 539)	*p* for gender
**Socio-demographic variables**				
School				
Urban area	47.4	51.5	44.5	0.039
Rural area	52.6	48.5	55.5	
Grade				
Seven	44.4	46.6	42.9	0.207
Eight	40.2	40.4	40.1	
Nine	15.4	12.9	17.0	
Smartphone ownership	64.1	68.5	61.0	0.022
**Social networking characteristics**				
Device used for social networking				
Personal computer	62.1	55.5	66.6	0.003
Smartphones	24.0	28.8	20.6	
Others (e.g. Ipad)	13.9	15.6	12.8	
Top three purposes for using social networking				
Keep in contact with old friends	73.3	76.4	71.1	—
Entertainment	48.3	49.5	47.7	
Make new friends	39.8	41.2	38.8	
Duration of social networking use				
<3 months	19.1	18.1	19.9	0.580
3–6 months	6.9	6.5	7.2	
7–12 months	8.0	6.7	8.9	
1–2 years	26.6	28.6	25.2	
>2 years	39.3	40.2	38.8	
Number of days/week				
≤1 day	29.0	25.3	31.5	0.119
2–3 days	46.8	51.2	43.8	
4–5 days	10.7	10.8	10.6	
≥6 days	13.5	12.7	14.1	
Amount of time/day				
<10 mins	20.0	14.3	23.9	<0.001
11–30 mins	30.3	31.8	29.3	
31–60 mins	28.1	33.2	24.7	
>60 mins	21.5	20.8	22.1	
Number of social networking friends				
<50	45.9	44.7	46.8	0.325
51–100	25.3	25.1	25.4	
101–200	17.8	20.8	15.8	
201–400	7.3	6.2	8.0	
>400	3.7	3.2	4.1	
**Social networking-related scales**				
SNAIS	23.4±10.2	24.4±9.3	22.6±10.7	0.006
SFUI	15.9±8.2	17.5±7.5	14.7±8.4	<0.001
EFUI	7.5±3.1	7.0±2.7	7.9±3.2	<0.001
Emotional connection to social networking	14.4±4.9	14.4±4.7	14.3±5.1	0.838
Social networking addiction	17.7±6.0	17.5±5.9	17.8±6.0	0.394
**Internet addiction (%)**	8.8	4.6	11.7	<0.001

*p* values for gender were obtained by applying Chi-square test to categorical variables and student *t*-test for continuous variables.

### Other variables used for validation

#### Characteristics related to social networking use

Participants were asked whether they currently possess any social networking account. Those providing an affirmative answer to the question were asked about their experiences in social networking use, including duration of social networking use, number of days per week on average using social networking, amount of time spent on social networking in a typical day, and number of social networking friends.

#### Emotional connection to social networking

Emotional connection to social networking was measured by six attitudinal items adapted from the Facebook Intensity Scale. [17] The Facebook Intensity Scale was developed to measure Facebook usage by incorporating both emotional connectedness to the site and its integration into one’s daily activities. In the present study, the word “Facebook” was replaced by “online social networking” for assessing extent of emotional connection. It was translated to English and back-translated to Chinese. Five-point Likert scales were used, ranging from 1 (strongly disagree) to 5 (strongly agree). Higher scores indicated a higher level of emotional connection to social networking. In our sample, an exploratory factor analysis revealed a one-factor solution for this scale, explaining 60.0% of the total variance, and the factor loadings of the six items ranged from 0.73 to 0.85. The Cronbach’s alpha coefficient was 0.87, and the test-retest intra-class correlation coefficient was 0.78.

#### Social networking addiction

Social networking addiction was measured by adapting items of the Facebook Addiction Scale, [28] which included eight items describing addictive symptoms: cognitive and behavioral salience, conflict with other activities, euphoria, loss of control, withdrawal, and relapse and reinstatement. Similarly, the word “Facebook” was replaced by “online social networking” in the current study, and a translation and back-translation process was used. Response categories rated from 1 (not true) to 5 (extremely true), with a higher score indicating a higher level of addictive tendency to social networking. In this study, an exploratory factor analysis of this scale showed a one-factor solution that explained 51.2% of the total variance, with high factor loadings ranging from 0.64 to 0.77. The Cronbach’s alpha coefficient was 0.86 and the test-retest intra-class correlation coefficient was 0.84.

#### Internet addiction

Internet addiction was measured by the 8-item Young’s Diagnostic Questionnaire (YDQ). It measures the degree to which the Internet affects different aspects of one’s daily life. Participants who provided five or more “yes” answers were classified as an Internet addiction case. [29,30] The scale has commonly been used in Chinese student populations and shown acceptable validity and reliability. [31,32] In our sample, the exploratory factor analysis revealed a one-factor solution for this scale, explaining 37.8% of the total variance, and the factor loadings ranged from 0.46 to 0.69. The scale showed good internal reliability (Cronbach’s α = 0.73).

### Statistical analysis

Descriptive statistics (e.g. means, standard deviation, and percentage) were presented when appropriate. Floor and ceiling effects were considered present if more than 15% of respondents possessed the minimum or maximum score of the SNAIS. [33] Internal consistency was assessed by Cronbach’s alpha coefficient, while test-retest reliability was evaluated by Intra-class Correlation Coefficient (ICC). Pearson correlation coefficients between the items and the Overall scale, between the items and their corresponding subscales, and between the items and the other subscales of the SNAIS were calculated for the item analysis. Besides, Pearson correlation coefficients among SNAIS/subscales, emotional connection to social networking, social networking addiction, and Internet addiction were derived. Furthermore, relationships between the SNAIS/subscales and general characteristics related to social networking use were tested by both Spearman correlation coefficients and one-way ANOVA to establish external validity.

To examine the factor structure of the SNAIS, the sample was randomly split into two subsamples of equal size. In the first subsample, the factor structure of the SNAIS was extracted by exploratory factor analysis (EFA) using principal components extraction method and varimax rotation methods. In the second subsample, confirmatory factor analysis (CFA) with maximum likelihood estimation was conducted to cross-validate the factor structure which was suggested by the EFA. The following goodness of fit statistics and cutoff criteria were used to evaluate the factor structure model in the CFA: [34–36] χ^2^/df ratio<5.00, both Non-Normed Fit Index (NNFI) and Comparative Fit Index (CFI) >0.95, and Root Mean Square Error of Approximation (RMSEA) <0.08. The confirmatory factor analysis was conducted by using Lisrel 8.70, while all other statistical analyses were performed by using SAS version 9.3 (SAS Institute, Cary, NC, USA). The statistical significant level was 0.05.

## Results

### Sample characteristics

Of the 910 participants, 44.4%, 40.2% and 15.4% were 7^th^, 8^th^ and 9^th^ grade students, respectively and 47.4% came from the urban school. About 60% of the participants were male (59.2%) and about two thirds (64.1%) of them possessed a smartphone (Table 1). The participants performed online social networking mainly via personal computers (62.1%) and smartphones (24.0%). The top three purposes for using online social networking were to keep in contact with old friends (73.3%), entertainment (48.4%), and to make new friends (39.8%). Over 65.0% had used online social networking services for more than one year, and 11.0% of the social networking users were daily users in the last month. On the days they reported using social networking, 49.7% spent on average more than 30 minutes per day for social networking. Furthermore, 8.8% of the participants were classified as having Internet addiction (12.0% for males and 4.6% for females, *χ*^2^ = 13.84, df = 1; *p*<0.001) (Table 1).

### Factor structures

The EFA revealed two factors with eigenvalue larger than one. The first 10-item factor explained 42.2% of the total variance, and was named the “Social Function Use Intensity (SFUI)” subscale. The second 4-item factor explained an additional 8.7% of the total variance, and was named the “Entertainment Function Use Intensity (EFUI)” subscale. All factor loadings exceeded 0.50 (Table 2), indicating that all items should be included in the confirmatory factor analysis. [37] The results of the CFA confirmed the two-factor solution, showing acceptable goodness of fit to the data: χ^2^/df = 4.05 (*χ*^2^ = 307.68, df = 76, *p*<0.001), NNFI = 0.96, CFI = 0.96, RMSEA = 0.082 (90%CI: 0.073~0.092). The standardized path estimates were above 0.48 (ranged from 0.48~0.76) (Table 2).

**Table 2:** Factor loadings and path loadings estimated by exploratory and confirmatory factor analyses for the Social Networking Activity Intensity Scale (SNAIS).

How often have you performed the following online social networking activities in the last month?		Factor loading(EFA)		Path estimate(CFA)
		SFUI	EFUI	
1	Sent messages to friends on message board	**0.71**	0.15	0.63
2	Chatted with friends via instant messaging function	**0.54**	0.38	0.67
3	Replied to comments made by social networking friends	**0.70**	0.21	0.74
4	Commented on friends’ status, logs, and photos	**0.80**	0.08	0.76
5	Shared/Forwarded content	**0.72**	0.12	0.73
6	Browsed others’ logs/photos/statuses/albums	**0.74**	0.20	0.72
7	Updated self-status	**0.66**	0.40	0.70
8	Posted photos/videos on personal web profile	**0.56**	0.26	0.59
9	Wrote logs/weibo	**0.66**	0.29	0.65
10	Decorated personal web profile(changed image/contact information/privacy setting)	**0.55**	0.46	0.71
11	Surfed entertainment/current news	0.20	**0.61**	0.49
12	Watched video/listened to music	0.34	**0.53**	0.63
13	Played games/applications	-0.02	**0.80**	0.49
14	Bought/gave virtual goods (e.g. birthday gifts)	0.24	**0.57**	0.48
	**Eigen value**	**5.91**	**1.22**	**-**
	**Cumulative % of Variance explained**	**42.21**	**50.93**	**-**

Note: SFUI: Social Function Use Intensity Subscale; EFUI: Entertainment Function Use Intensity Subscale; EFA: Exploratory Factor Analysis; CFA: Confirmatory Factor Analysis.

### Ceiling and floor effects of the SNAIS and its two subscales

The percentage of respondents reporting maximum scores of the SFUI, EFUI, and SNAIS were 0.88%, 0.66% and 0.55%, respectively, while the percentage of participants reporting minimum scores of the SFUI, EFUI and SNAIS were 0.88%, 1.76% and 0.55%, respectively. Therefore, no significant ceiling or floor effects were observed for the SNAIS and its two subscales.

### Item analysis

Except for Item 8 and Item 14, item means were close to the middle of the range. The correlation coefficients between each item and the overall scale ranged from 0.43 to 0.72 (all *p*<0.001), while all correlation coefficients between the items and their corresponding subscale ranged from 0.64 to 0.77 (all *p*<0.001). All correlation coefficients between individual items and their respective subscale were higher than those between the same items and the other subscale (Table 3).

**Table 3:** Item analysis of the SNAIS (*n* = 910).

Item	$\bar{X}\pm SD$	Cronbach's alpha if item is deleted		Item-total correlation	Item-subscale correlation	Item-another subscale correlation
		Subscale	Total			
1	1.34±1.02	0.89	0.88	0.66^‡^	0.69^‡^	0.34^‡^
2	2.24±1.11	0.89	0.88	0.68^‡^	0.68^‡^	0.45^‡^
3	1.59±1.23	0.88	0.88	0.72^‡^	0.75^‡^	0.37^‡^
4	1.50±1.14	0.88	0.88	0.73^‡^	0.77^‡^	0.35^‡^
5	1.76±1.25	0.89	0.88	0.70^‡^	0.74^‡^	0.36^‡^
6	1.87±1.15	0.88	0.88	0.72^‡^	0.75^‡^	0.40^‡^
7	1.78±1.13	0.88	0.88	0.74^‡^	0.75^‡^	0.46^‡^
8	0.88±1.02	0.89	0.88	0.61^‡^	0.64^‡^	0.32^‡^
9	1.29±1.19	0.89	0.88	0.70^‡^	0.71^‡^	0.40^‡^
10	1.61±1.09	0.89	0.88	0.72^‡^	0.72^‡^	0.46^‡^
11	1.72±1.10	0.56	0.89	0.49^‡^	0.64^‡^	0.38^‡^
12	2.69±1.09	0.52	0.89	0.57^‡^	0.68^‡^	0.45^‡^
13	2.22±1.25	0.49	0.89	0.43^‡^	0.74^‡^	0.26^‡^
14	0.87±1.07	0.56	0.89	0.51^‡^	0.64^‡^	0.39^‡^

Note: Item-total correlation: Spearman correlation coefficient between each item and the Overall scale. Item-subscale correlation: Spearman correlation coefficient between each item and its corresponding subscale. Item-another subscale correlation: Spearman correlation coefficient between each item and the other subscale.

^‡^: *p*<0.001

### Reliability

Internal reliability based on the entire sample was acceptable for the SNAIS (Cronbach’s alpha = 0.90, 0.60, and 0.89 for the SFUI, EFUI, and SNAIS, respectively). The test-retest intra-class correlation coefficients were 0.87, 0.67, and 0.85 for two subscales and the Overall scale, respectively.

### Correlation between SNAIS, emotional connection to social networking, social networking addiction, and Internet addiction

The scores of the SNAIS and its two subscales were positively correlated with the scales of emotional connection to social networking and social networking addiction. The SFUI showed slightly stronger correlations with emotional connection (Pearson *r* = 0.49, *p*<0.001) and social networking addiction (Pearson *r* = 0.32, *p*<0.001), as compared to those of the EFUI (Pearson *r* = 0.40 and 0.25, respectively, *p*<0.001). In contrast, the EFUI presented slightly stronger correlations with Internet addiction (Pearson *r* = 0.23, *p*<0.001) as compared to that of the SFUI (Pearson *r* = 0.18, *p*<0.001) (Table 4).

**Table 4:** Correlations among SNAIS, emotional connection, social networking addiction and Internet addiction (*n* = 910).

	SNAIS			Emotional connection to social networking	Social networking addiction
	Overall	SFUI	EFUI		
SNAIS	**-**				
SFUI	0.97^‡^	**-**			
EFUI	0.74^‡^	0.54^‡^	**-**		
Emotional connection to social networking	0.52^‡^	0.49^‡^	0.40^‡^	**-**	
Social networking addiction	0.34^‡^	0.32^‡^	0.25^‡^	0.56^‡^	**-**
Internet addiction	0.22^‡^	0.18^‡^	0.23^‡^	0.38^‡^	0.49^‡^

SNAIS: Social Networking Activity Intensity Scale; SFUI: Social Function Use Intensity subscale; EFUI: Entertainment Function Use Intensity subscale

^‡^: *p*<0.001

### Relationship between SNAIS and participants’ characteristics of social networking use

Duration of social networking use, number of days per week on average using social networking, amount of time per day on average spent on social networking, and number of social networking friends were all positively correlated with the SFUI (Spearman *r* = 0.26 to 0.38, all *p*<0.001), EFUI (Spearman *r* = 0.27 to 0.30, all *p*<0.001), and SNAIS (Spearman *r* = 0.30 to 0.40, all *p*<0.001) (Table 5).

**Table 5:** Spearman correlations between SNAIS scores and characteristics of social networking use (*n* = 910).

	SNAIS	SFUI	EFUI
Duration of social networking use	0.30^‡^	0.26^‡^	0.28^‡^
Number of days/week	0.36^‡^	0.34^‡^	0.29^‡^
Amount of time/day	0.32^‡^	0.38^‡^	0.27^‡^
Number of social networking friends	0.40^‡^	0.38^‡^	0.30^‡^

SNAIS: Social Networking Activity Intensity Scale; SFUI: Social Function Use Intensity subscale; EFUI: Entertainment Function Use Intensity subscale

^‡^: *p*<0.001

Moreover, the results of the one-way ANOVA analysis showed that those who reported higher levels of duration of social networking use, number of days per week on average using social networking, amount of time spent on social networking in a typical day, and number of social networking friends, scored significantly higher in the SNAIS and its two subscales (all *p*<0.001, Table 6).

**Table 6:** Mean differences of scores of SNAIS, SFUI and EFUI by groups (*n* = 910).

	%	SNAIS		SFUI		EFUI	
		$\bar{X}\pm SD$	*p*	$\bar{X}\pm SD$	*p*	$\bar{X}\pm SD$	*p*
Duration of social networking use							
<3 months	19.1	17.8±9.6	<0.001	11.6±7.7	<0.001	6.2±2.9	<0.001
3–6 months	6.9	21.6±7.9		14.7±6.7		7.0±2.5	
7–12 months	8.0	21.8±9.2		15.0±7.3		6.8±2.8	
1–2 years	26.6	23.7±9.4		16.3±7.7		7.4±2.6	
>2 years	39.3	26.4±10.3		18.0±8.4		8.4±3.3	
Number of days/week							
≤1 day	29.0	17.8±9.4	<0.001	11.5±7.7	<0.001	6.3±2.9	<0.001
2–3 days	46.8	24.6±8.8		17.0±7.1		7.6±2.8	
4–5 days	10.7	25.0±9.2		17.2±7.7		7.8±2.8	
≥6 days	13.5	29.6±11.4		22.2±9.3		9.4±3.3	
Amount of time/day							
<10 mins	20.0	17.3± 9.2	<0.001	10.9±7.3	<0.001	6.4±3.0	<0.001
11–30 mins	30.3	21.7± 8.2		14.7±6.8		7.0±2.6	
31–60 mins	28.1	25.2± 9.2		17.5±7.4		7.7±2.9	
>60 mins	21.6	28.9±11.3		20.0±9.0		9.0±3.3	
Number of social networking friends							
<50	45.9	19.5± 9.1	<0.001	12.9±7.4	<0.001	6.6±2.8	<0.001
51–100	25.3	23.9± 8.5		16.3±6.9		7.7±2.8	
101–200	17.8	27.0± 9.6		18.7±7.9		8.3±2.8	
201–400	7.3	31.3±10.0		21.8±8.1		9.5±3.3	
>400	3.7	33.8±13.2		24.1±10.3		9.7±4.3	

Note: SNAIS: Social Networking Activity Intensity Scale; SFUI: Social Function Use Intensity Subscale; EFUI: Entertainment Function Use Intensity Subscale

*p* values were obtained by one-way ANOVA

^‡^: *p*<0.001

## Discussion

In the present study, we have developed and validated the Social Networking Activity Intensity Scale (SNAIS) among junior middle school students in China. To our knowledge, this is the first study to develop a tool that assesses online social networking use intensity (SNUI) among adolescents based on diverse social networking activities. The results showed that the SNAIS had acceptable psychometric properties. Considering the dramatically increasing prevalence of social networking use among adolescents (90% prevalence in our sample), the development of the SNAIS would facilitate the progression of related studies investigating the effects of social networking use on psychosocial well-being among adolescents.

The developed SNAIS is a comprehensive inventory with acceptable psychometric properties. We found acceptable internal and test-retest reliability of SNAIS. The test-retest findings demonstrated that the SNAIS and its two subscales were stable over time. Furthermore, there were no noticeable ceiling or floor effects.

The results being that the correlations between items and their hypothesized subscales were high but those between items and other subscales were weak indicated good content validity for social function and entertainment function assessment related to social networking use. The factor structure of SNAIS was also clearly established. Results of factor analysis yielded a two-factor solution (Social Function Use Intensity and Entertainment Function Use Intensity), which reflects two different but important behavioral patterns related to social networking activities among adolescents. It is encouraging that the two identified factors of the SNAIS were positively correlated with traditional measures related to social networking characteristics (i.e. duration of social networking use, number of days per week using social networking on average, time spent on social networking on a typical day, and number of social networking friends). Moreover, based on the results of one-way ANOVA, individuals who reported longer duration use, more frequent use, longer time spent in a typical day, and having more friends on online social networking were found to have higher scores of SNAIS and its two subscales as compared to those in the corresponding lower categories. The findings suggested that SNAIS could successfully discriminate intensive users from infrequent users. Thus, it could assess individual social networking use intensity more thoroughly than those simple questions.

The SNAIS that was developed in the present study provided a new angle to measure online social networking use intensity as compared to those previous scales. For instance, FIS, one of the scales commonly used in previous studies, only applied two items to assess Facebook behaviors (i.e. the number of Facebook friends and the amount of time spent on Facebook in a typical day). The single item related to intensity of use is inadequate and no testing of psychometric properties such as factor structure and internal reliability was possible. It is also confined to a single platform (Facebook). Conceptually, FIS mainly evaluated the individual’s attitudes towards Facebook to reflect the degree of emotional connection to Facebook. In contrast, the SNAIS mainly focused on the online social networking activities instead of confining to only one specific platform (e.g. Facebook). It assessed social networking use intensity for specific functions, in which two constructs (social function and entertainment) emerged. The development of SNAIS therefore adds value to existing tools. As it was developed to assess social networking use intensity, it does not include other constructs such as emotional attachment. Such a limitation could be addressed in future revision of the scale.

The two subscales that emerged from the SNAIS have strong practical implications and can be used to understand various consequences of SNUI. Previous research has indicated that the effects of Internet use on psychosocial well-being was partially dependent on the nature of Internet use.[38] A one-year longitudinal study revealed that Internet use for communication purposes was protective of depression, while the use for non-communicative purposes predicted depression and social anxiety.[39] The two constructs of the SNAIS would hence facilitate future investigations of variations between effects of the two main types of social networking use on mental health and other outcomes among adolescents.

Discussion is warranted for some findings. Firstly, the EFUI subscale gave acceptable but relatively low internal (α = 0.60) and test-retest reliability (ICC = 0.67), as compared to that of the SFUI subscale. Adding additional items to the EFUI subscale may improve its reliability. Furthermore, the specific entertainment functions included in the subscale (e.g. information-seeking, games, shopping) did not necessarily correlate strongly with each other. Further elaborations and refinements are warranted. We believe that the EFUI subscale is still useful, considering its acceptable psychometric properties. We suggest both subscales to be used together in the future, as nowadays social interactions and entertainment are not mutually exclusive. Future revision of the scale may modify some items of EFUI to improve its internal reliability. Furthermore, the results showed that the EFUI had a slightly higher correlation with Internet addiction as compared with that of the SFUI. Such an observation needs to be confirmed in the future research.

The present study has several limitations. Firstly, data was acquired by self-reporting collection method, reporting bias might exist although the study was highly anonymous. Secondly, participants were conveniently recruited from only two junior middle schools in one city (i.e. Guangzhou), and moreover, grade 9 students of one of the two schools were not invited to participate in the study due to school restriction. Multi-stage cluster sampling may be a better study design, and may require a larger sample size; future studies should use this design. The convenient sample method might cause selection bias, but as we studied factor structures, we believe that the bias would be smaller than that of prevalence/risk factors estimations. We followed the study design of a number of published papers involving validation of scales among students [40–42], which have smaller sample sizes as compared to our study. Therefore, the psychometric properties of SNAIS should be evaluated in a large representative sample in the future, as Internet penetration and behavioral patterns of social networking use may vary across geographic regions in China. Thirdly, nature of online social networking use has been evolving rapidly, therefore new activities and functions of social networking might need to be considered in the future.

In sum, the study developed and evaluated an instrument for assessing extensive functions of social networking use intensity. It has acceptable psychometric properties and is easy to be self-administered by junior middle school students. Its application may catalyze the development of relevant research on the impact of social networking use among adolescents in China and beyond.
